# E-Cigarettes Increase *Candida albicans* Growth and Modulate its Interaction with Gingival Epithelial Cells

**DOI:** 10.3390/ijerph16020294

**Published:** 2019-01-21

**Authors:** Humidah Alanazi, Abdelhabib Semlali, Witold Chmielewski, Mahmoud Rouabhia

**Affiliations:** Groupe de Recherche en Écologie Buccale, Faculté de Médecine Dentaire, Université Laval, 2420 de la Terrasse, Québec, QC G1V 0A6, Canada; humidah.alanazi.1@ulaval.ca (H.A.); abdelhabib.semlali.1@ulaval.ca (A.S.); Witold.Chmielewski@fmd.ulaval.ca (W.C.)

**Keywords:** e-cigarettes, *C. albicans*, growth, *SAP* genes, epithelial cells, LDH

## Abstract

Electronic cigarette (e-cigarette) vapor comes in contact with the different constituents of the oral cavity, including such microorganisms as *Candida albicans*. We examined the impact of e-cigarettes on *C. albicans* growth and expression of different virulent genes, such as secreted aspartic proteases (*SAPs*), and the effect of e-cigarette vapor-exposed *C. albicans* on gingival epithelial cell morphology, growth, and lactate dehydrogenase (LDH) activity. An increase in *C. albicans* growth was observed with nicotine-rich e-cigarettes compared with non-exposed cultures. Following exposure to e-cigarette vapor, *C. albicans* produced high levels of chitin. E-cigarettes also increased *C. albicans* hyphal length and the expression of *SAP2*, *SAP3*, and *SAP9* genes. When in contact with gingival epithelial cells, e-cigarette-exposed *C. albicans* adhered better to epithelial cells than the control. Indirect contact between e-cigarette-exposed *C. albicans* and gingival epithelial cells led to epithelial cell differentiation, reduced cell growth, and increased LDH activity. Overall, results indicate that e-cigarettes may interact with *C. albicans* to promote their pathogenesis, which may increase the risk of oral candidiasis in e-cigarette users.

## 1. Introduction

Cigarette smoking constitutes a well-established risk factor for oral infections [1]. Indeed, smokers are more prone to severe periodontal disease, caries, and candidoses [2,3]. Data have shown that cigarette smoke extract alters the interaction between *Porphyromonas gingivalis* and the host, leading to periodontitis [4]. Although periodontitis has been strongly associated with bacteria such as *P. gingivalis*, a variety of microorganisms, including *Candida albicans*, have been detected in periodontal pockets [5,6]. *C. albicans* has thus been associated not only with periodontitis, but also with oropharyngeal candidoses [5,7]. Patients with systemic disorders such as diabetes mellitus, neutropenia, agranulocytosis, and acquired immunodeficiency syndrome (AIDS) have also been shown to harbor enteric *Staphylococcus aureus* and *Candida* sp. in their periodontal pockets [6,8]. Furthermore, studies have reported the presence of *C. albicans* in non-immunologically compromised patients suffering from severe chronic periodontitis [5,9].

*Candida* virulence was promoted by various exogenous factors, such as cigarette smoke [3], which has been shown to stimulate *C. albicans* adhesion and growth, as well as biofilm formation [3,10]. Conventional cigarette smoke (CCS) was also found to promote *C. albicans* growth, with an increased expression of enhanced adherence to polystyrene (*EAP1*), hyphal wall protein 1 (*HWP1*), and certain secreted aspartyl proteinase (*SAP*) genes known to be involved in the yeast’s virulence [10].

To counter the adverse effects of cigarette smoke, an electronic cigarette (e-cigarette) was introduced on the market and promoted as a “safe alternative” to the smoking habit [11]. The e-cigarette combines a plastic tube, an electronic heating component, and a reservoir for an e-liquid solution that contains propylene glycol and glycerol, with or without nicotine [12]. Following airflow detection by the internal sensor in the e-cigarette device, the heating component in contact with the e-liquid produces a vaping solution of a smoke-like aerosol that is subsequently inhaled into the upper airways [12].

During e-cigarette use, the first site in contact with the e-liquid vapor is the oral cavity, including the gingival tissues and the oral microbial community. E-cigarette use reportedly induces harmful free radicals and inflammation leading to gingival cell damage, which may affect the innate defense, thereby promoting oral infections [13].

As the effect of e-cigarettes on oral microorganisms such as *C. albicans* has not yet been fully elucidated, we sought to analyze the growth and expression of the *SAP2*, *SAP3* and *SAP9* genes by *C. albicans* following multiple exposures to conventional cigarette smoke (CCS), nicotine-rich (NR) e-cigarettes, and nicotine-free (NF) e-cigarettes. We also investigated the interaction between e-cigarette-exposed *C. albicans* and gingival epithelial cells.

## 2. Materials and Methods

### 2.1. Candida Strain

*C. albicans* (ATCC-SC5314) was grown in Sabouraud liquid medium (Becton Dickinson, Cockeysville, MD, USA) supplemented with 0.1% glucose. The culture was grown to the stationary phase for 18 h at 30 °C in a shaking water bath. The blastoconidia were collected, washed with phosphate-buffered saline (PBS), and counted by means of a hemacytometer (Reichert, Buffalo, NY, USA). The cell suspension was adjusted to 10^8^ *C. albicans* cells/mL prior to being exposed or not to CCS or e-vapor.

### 2.2. E-Cigarettes

eGo ONE CT electronic cigarette devices (www.joyetech.com) purchased from local retailers (Québec City, QC, Canada) were used to deliver the e-cigarette vapor. There are three modes of eGo ONE CT: CT-Ti (Titanium), CT-Ni (Nickel 200), and CW. The CW mode refers to 25 W/15 W/7.5 W, with a 1100 mAh battery. The eGo e-cigarette device has a 1.8 mL tank atomizer, as specified by the manufacturer.

Disposable e-cigarette liquids with and without nicotine (flavor: Smooth Canadian tobacco, http://shop.juicyejuice.com/juicy-canadian-tobacco-e-liquid.ejuice) were included in this study. The e-liquids (with and without nicotine) contained about 70% propylene glycol, 30% vegetable glycerin, and natural and artificial food grade flavoring as specified by the manufacturer. The nicotine concentration in the e-liquid was 18 mg/mL. The selected e-cigarette devices and e-liquids were chosen because of their availability to users. For the conventional cigarette, we used 1R3F cigarettes purchased from the Kentucky Tobacco Research & Development Center (Orlando, FL, USA).

### 2.3. Effect of e-Vapor on C. albicans Growth

**Figure 1:**
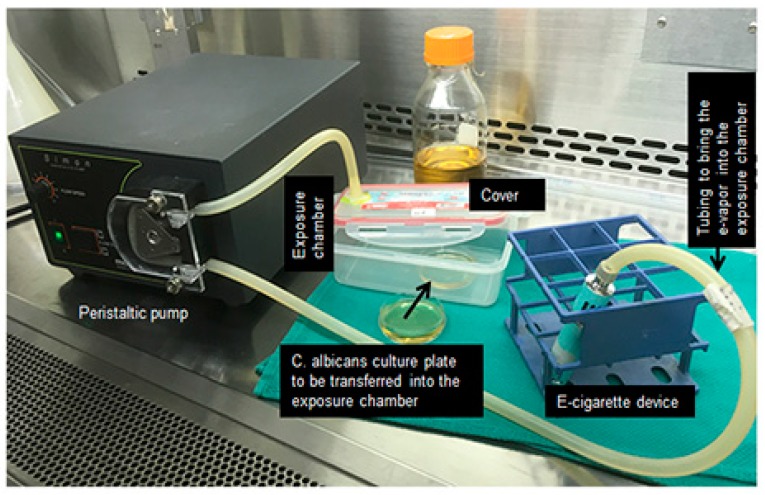
Exposure protocol of *Candida albicans* to e-cigarette vapor or combustible cigarette smoke.

*C. albicans* (10^6^ cells) were placed in a 50 mL sterile culture tube containing 2 mL of fresh Sabouraud liquid medium. The following four conditions were used in each *C. albicans* culture experiment: Non-exposed to CCS, exposed to CCS, NR e-vapor, or NF e-vapor. The exposures to the e-cigarettes vapor were performed using a peristaltic pump and custom-made smoke chambers (see Figure 1). Briefly, *C. albicans* cultures in 60 mm diameter Petri dishes were aseptically placed inside the smoke chamber. The e-cigarette device was linked to one end of a silicone tube while the other end of the tube was linked to the smoke chamber. The peristaltic pump was used to deliver the e-cigarette vapor into the chamber. Following activation of the peristaltic pump, the e-cigarette device delivered the e-cigarette vapor through the silicone tube into the exposure chamber. The e-vapor (with and without nicotine) drawn into the chamber represented 2 puffs every 60 s with a 4 to 5 s puff followed by a 25 to 30 s pause, as previously described [14], with some modifications. With this procedure, *C. albicans* cells were atmospherically exposed to the e-vapor. To promote contact of *C. albicans* cells with e-vapor, the cultures were gently agitated during and after each puff. The exposure procedure to CCS was identical to that used with the e-vapor. Briefly, a cigarette was linked to one end of a silicone tube while the other end of the tube was linked to the smoke chamber. The peristaltic pump allowed for the delivery of CCS equivalent to half a cigarette into the chamber, with an approximate 20 s burning time. For each condition, the exposure time consisted of 15 min twice a day for 2 and 3 days. The *C. albicans* cells were incubated at 37 °C, between and after exposures to e-cigarettes. The used e-liquid volume during the 15 min exposure was 350 μL.

Each *C. albicans* exposure condition was subsequently incubated for one additional hour prior to medium changing. The *C. albicans* pellets were then fed fresh Sabouraud medium and were cultured until the following exposure, prior to undergoing various analyses. *C. albicans* cultures were placed into a smoke-free/e-vapor-free chamber for the same duration as the assay conditions that were included in the study as controls (Ctrl). Each exposure condition (CCS, NF, NR, and Ctrl) was performed in a separate exposure chamber to avoid culture cross-contamination. At the end of each exposure regime (2 or 3 days), *C. albicans* growth was determined by MTT assay, as previously reported [15]. Results were reported as means ± SD, *n* = 5.

### 2.4. Effect of e-Vapor on C. albicans Cell Wall Chitin Content

In the first set of experiments, *C. albicans* cells (10^6^) were seeded in Sabouraud liquid medium and exposed twice a day to CCS, NF e-vapor, or NR e-vapor for 15 min each, and were cultured thereafter for 24 h at 37 °C. The cells were then collected and centrifuged, with the resulting pellets suspended in 500 μL of 4% paraformaldehyde solution to fix the cells. After 60 min of incubation at room temperature, the cells were washed twice with PBS and incubated thereafter for 5 min at room temperature with calcofluor white stain in the presence of 10% potassium hydroxide. The cells were then observed under an epifluorescence (UV) microscope and photographed.

In the second set of experiments, *C. albicans* cells exposed twice a day to CCS, or NF or NR e-vapor for 15 min and cultured for 16 h at 37 °C were then collected and centrifuged, with the pellets subsequently suspended in 2 mL of fresh Sabouraud medium. The cell count was initiated on each condition using a hemocytometer counting protocol. An exact amount of *C. albicans* cells (400 × 10^6^) from each condition (CCS, NR, NF, and Ctrl) was then centrifuged, with the resulting pellet resuspended in 700 μL of lysis buffer containing 200 μL of glass beads (0.425–0.6 mm in diameter). The cells were disrupted by means of a MiniBead-beater (Biospec Products, Bartlesville, OK, USA) for 2 min at 5000 rpm for 10 cycles under cold conditions [16]. Each cell wall was hydrolyzed in 6N HCl for 16 h at 100 °C, evaporated at 65 °C, and dissolved thereafter in sterile water (1 mL). A small volume (100 μL) of each solution was supplemented with 100 μL of 1.5N Na_2_CO_3_ in 4% acetylacetone and boiled (100 °C) for 30 min, after which 0.7 mL of 96% of ethanol was added. A volume of 100 μL of a solution containing 1.6 g of 4-(dimethylamino)benzaldehyde (Sigma Aldrich, Oakville, Ontario) in 30 mL of HCl and 30 mL ethanol was added to each sample, with the resulting mixture incubated thereafter for 1 h at 37 °C in a dark atmosphere. Absorbance was measured at 520 nm by means of an xMark microplate spectrophotometer (Bio-Rad, Hercules, CA, USA). Chitin concentrations were calculated using a standard curve of glucosamine (0–200 mg, *n* = 4).

### 2.5. Effect of e-Vapor on C. albicans Transition from Blastospore to Hyphal Form

Qualitative and quantitative assays were performed to determine the impact of e-cigarette vapor on *C. albicans* morphological changes (yeast to hyphae). *C. albicans* (10^5^ cells) were grown in 3 mL of Sabouraud liquid medium supplemented with 0.1% glucose and 10% fetal bovine serum (FBS, Burlington, ON, Canada). The cultures were immediately exposed to CCS, NR e-vapor, or NF e-vapor for 15 min, followed by incubation for either 3 or 6 h at 37 °C prior to assessment of cell morphological changes. CCS was considered as the positive control, while non-exposed *C. albicans* cultures were considered as the negative controls. Following incubation for 3 or 6 h, cultures were observed microscopically and photographed to record the *C. albicans* morphology (*n* = 5), while hyphal length in each condition was measured by means of NIH-ImageJ software (Version 1.52j).

### 2.6. Effect of e-Vapor on the Expression of SAP2, SAP3, and SAP9 genes by C. albicans

*C. albicans* (5 × 10^6^ cells) were first placed in a 50 mL sterile culture tube containing 2 mL of fresh Sabouraud liquid medium. The cells were then exposed or not to CCS, NR e-vapor, or NF e-vapor twice for 15 min, with a 6 h interval between each exposure. Exposure to CCS and e-cigarettes was performed using a peristaltic pump and a smoke chamber. Following each exposure, cultures were incubated for 60 min before the culture medium was refreshed. Following the second exposure, *C. albicans* cultures were incubated for 16 h at 37 °C and subsequently used to extract total RNA, as we previously reported [15]. The RNA (1 μg of each sample) was reverse transcribed into cDNA by means of the iScript cDNA Synthesis kit (Bio-Rad) and used for quantitative PCR (qPCR). Reactions were performed using a PCR supermix (Bio-Rad; iQ SYBR Green supermix). Specific *SAP2*, *SAP3*, and *SAP9* primers (Table 1) were added to the reaction mix at a final concentration of 250 nmol/L. Five microliters of each cDNA sample were added to a 20 μL PCR mixture containing 12.5 μL of the iQ SYBR Green supermix, 0.5 μL of each primer (*ACT1*—housekeeping gene, *SAP2*, *SAP3*, and *SAP9*) along with 7 μL of RNase/DNase-free water. Reactions were performed using a Bio-Rad MyCycler Thermal Cycler. The CT was automatically determined using the accompanying Bio-Rad CFX Manager. The thermocycling conditions for each gene were established as 5 min at 95 °C, followed by 30 cycles of 15 s at 95 °C, 30 s at 60 °C, and 30 s at 72 °C, with each reaction performed in triplicate. The specificity of each primer pair was determined by the presence of a single melting temperature peak. *ACT1* produced uniform expression levels, varying by <0.5 threshold cycles (CTs) between sample conditions, and thus became the reference gene for this study. The results were analyzed using the 2^−ΔΔCt^ (Livak) relative expression method [17] (*n* = 5).

### 2.7. Adhesion of e-Vapor-Exposed C. albicans to Gingival Epithelial Cells

Human gingival epithelial carcinoma cell line (Ca9-22), purchased from Health Science Research Resources Bank (HSRRB) (Osaka, Japan), was used for our in vitro experiments [18]. Cells were maintained in the Roswell Park Memorial Institute medium (RPMI)-1640 (Sigma-Aldrich Canada Cie. Oakville, ON), supplemented with L-glutamine and 10% fetal bovine serum (FBS). In a first set of experiments, we analyzed the adhesion of smoke- or vapor-exposed *C. albicans* to an epithelial cell monolayer. Briefly, *C. albicans* cells (10^6^) were exposed twice a day for 15 min with a 6 h interval between the first and second exposure to CCS, NR e-vapor, NF e-vapor, or nothing. Cultures were maintained at 37 °C for 24 h. The following day, smoke-exposed, vapor-exposed, or non-exposed *C. albicans* cultures were used to count the cell number, and then each one was used to infect the epithelial cell monolayers. For this purpose, Ca9-22 cells (10^5^) were seeded in 6-well tissue culture plates and incubated at 37 °C for 24 h before contact with *C. albicans*. The epithelial cell cultures were then pulsed with 10^4^ *C. albicans* that had or had not been exposed to CCS, NF, or NR products. The contact periods between epithelial cells and *C. albicans* were 6 and 24 h. At the end of each period, the medium was removed, the cultures were washed twice with fresh medium, with gentle agitation to remove non-adherent *C. albicans* cells. Following this, cultures were fixed with 4% paraformaldehyde for 60 min and stained with crystal violet dye. After staining, cultures were examined under an optical microscope and photographed. Each condition was run in triplicate (*n* = 4).

### 2.8. Growth of Epithelial Cells Following Indirect Contact with e-Vapor-Exposed C. albicans

Epithelial cells were seeded (10^5^) in 6-well tissue culture plates and cultured for 24 h at 37 °C in a 5% CO_2_ incubator. The following day, the culture medium was refreshed and e-vapor- and CCS-exposed *C. albicans* was put in contact with epithelial cells through a transwell culture system (PET membrane bottom, transparent, pore size 0.4 µm, Sarstedt Inc. Montreal, QC Canada). The *C. albicans* recipient well received 10^6^ cells. The porosity of the membrane was 0.4 um to allow for medium exchange but not *C. albicans*, which prevented the direct adverse effect of *C. abicans* on the epithelial cells. The transwell culture plates were then incubated at 37 °C in a 5% CO_2_ humid atmosphere for 24 h prior to analysis. The following day, the upper chamber was used to collect *C. albicans* cells, which were washed twice with Sabourand medium and then the cell number was counted to discriminate the blastospores and hyphal forms. The culture supernatants were collected and subjected to lactate dehydrogenase (LDH) cytotoxicity assay (Promega, Madison, WI, USA), as per the manufacturer’s protocol. For LDH activity measurement, we also included a positive control, which was obtained by incubating the gingival epithelial cells in the presence of 1% Triton X-100 (100% cell death); a negative control was obtained by culturing gingival epithelial cells under normal cell growth conditions. Epithelial cell shape was ascertained by inverted optical microscopy and was then photographed. Following this, epithelial cells were detached following incubation with a 0.05% trypsin-0.04% EDTA solution. Epithelial cell suspensions were used to determine the viable cell numbers in each condition, as determined by the trypan blue exclusion assay. The cell suspensions were then centrifuged, and the resulting cell pellets were lysed to extract total proteins to be used for subsequent analyses. Each experiment was performed in duplicate, and the means ± standard deviations of four separate experiments were calculated and plotted.

### 2.9. Statistical Analysis

Continuous variables were expressed using mean ± SD. Data were analyzed using a two-way ANOVA. CCS, NF, and NR-exposed, as well as non-exposed conditions, were applied for 15 min exposure or not to define four conditions. All of the statistical analyses had a significant interaction factor (*p* < 0.0001) and expressed heterogeneous variances. The Satterthwaite’s degree of freedom statement was added for unequal variance structures. Comparisons among the different conditions at different days (2 and 3 days for *C. albicans* growth analyses) were performed by partitioning the interactions. The normality assumption was verified using the Shapiro-Wilk test following a Cholesky factorization. Results were considered significant with *p* values ≤ 0.05. All of analyses were conducted using the SAS 9.4 statistical package (SAS Institute Inc., Cary, NC, USA) and R (R Core Team (2016), Foundation for Statistical Computing, Vienna, Austria).

## 3. Results and Discussion

### 3.1. E-Cigarette Vapor Promoted C. albicans Growth

E-cigarettes are proposed as a “safe alternative” to conventional cigarettes and a possible option to quit smoking [11]. As a result, the number of e-cigarette users and consumer acceptability has increased, despite false safety recommendations regarding this smoking process [19]. Indeed, data have unfortunately confirmed that e-cigarettes/e-vapors are not as safe as users believe them to be. Following use, e-cigarette vapor first comes in contact with the oral cavity, which may affect the oral tissue. Indeed, studies report a definite adverse effect of e-cigarettes on gingival cells [20].

**Figure 2:**
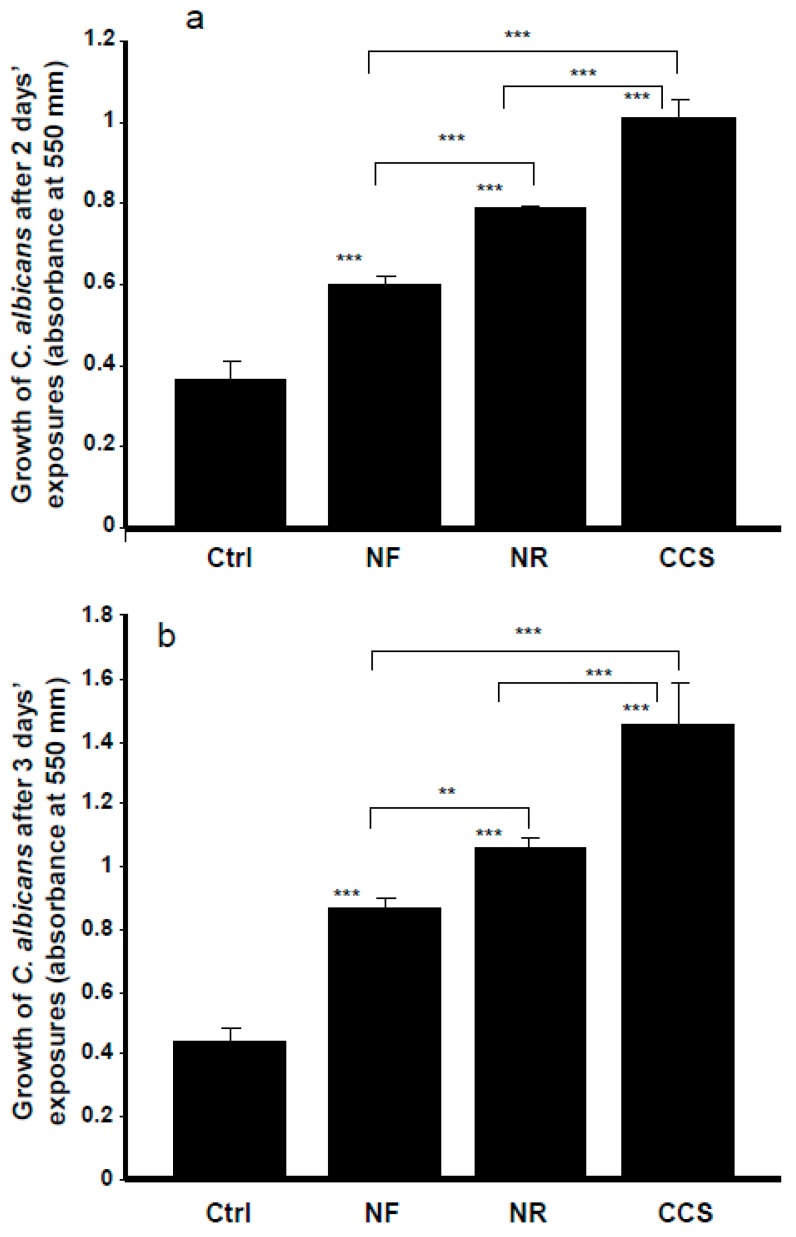
The growth of *C. albicans* was promoted by e-cigarette vapor. Cells were exposed or not for 15 min twice a day for 2 (panel **a**) or 3 (panel **b**) days, with the growth determined by MTT assay. Results are means ± SD (*n* = 5). A significant difference was observed when comparing the *C. albicans* cells exposed to CCS, NR e-vapor, or NF e-vapor and those of the control (non-exposed cells). We also compared NF to NR, NR to CCS, and NF to CCS. ** *p* < 0.01; *** *p* < 0.001. Free asterisks refer to the statistical difference when comparing exposed (e-vapors/CCS) with non-exposed (Ctrl) *C. albicans* cells. Bars with asterisks show the comparison of NF to NR, NR to CCS, and NF to CCS.

E-cigarettes could also modulate the oral microbial community. Our results indicate that e-cigarette vapor promoted *C. albicans* growth. Exposure of *C. albicans* cultures to NR e-cigarette vapor for 15 min twice a day over 2 days showed significantly (*p* < 0.001) increased *C. albicans* growth, compared to that observed in the controls (non-exposed cultures) (Figure 2). Following the MTT assay, the absorbance increased from 0.37 ± 0.04 in the control to 0.79 ± 0.003 in the presence of NR e-vapor. It should be noted that NF e-vapor also increased the growth of *C. albicans*, with its absorbance increasing from 0.37 ± 0.04 to 0.6 ± 0.02 (Figure 2). However, both the NR and NF e-vapor recorded low *C. albicans* growth, compared to that observed with CCS. For the NR e-vapor, the absorbance was 0.79 ± 0.003, while it was 1.015 ± 0.04 with CCS (Figure 2a). Comparable results were obtained after 3 days of exposure (Figure 2b), showing a significant (*p* < 0.001) growth increase when comparing the absorbance obtained with the NR or NF e-vapor and the control. A significant increase of *C. albicans* growth was also recorded when comparing CCS results and the NR and NF e-vapor results. It should also be noted that NR e-cigarettes significantly (*p* < 0.01) promoted *C. albicans* growth, compared to that recorded by the NF e-vapor.

### 3.2. Chitin Content was High in e-Cigarette Vapor-Exposed C. albicans

**Figure 3:**
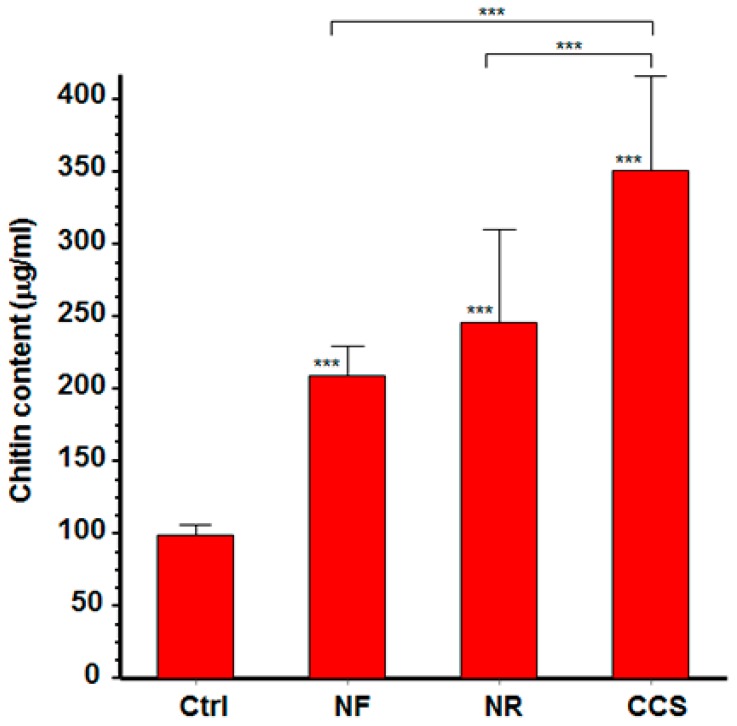
E-cigarette vapor increased the level of chitin produced by *C. albicans*. Following exposure or not to CCS, NR e-vapor, or NF e-vapor, cell wall proteins were extracted and subjected to chitin level quantification (see Materials and Methods section). Chitin levels are presented. Statistical significance was obtained by comparing the cells exposed to CCS or to NR or NF e-vapor and those of the control (non-exposed cells). *** *p* < 0.001; Free asterisks refer to the statistical difference when comparing exposed (e-vapors/CCS) to non-exposed (Ctrl) *C. albicans*. Bars with asterisks show the comparison of NR to CCS, and NF to CCS.

*C. albicans* growth following exposure to e-cigarette vapor was accompanied by increased chitin production. The fluorescence intensity of CCS-exposed and e-vapor-exposed cells was higher than that expressed by non-exposed cells. Cell density with intense fluorescence was also higher in CCS-exposed and e-vapor-exposed cultures than in non-exposed cultures (data not shown). The effect of e-vapor on chitin production was supported by the quantitative analyses of chitin content. Indeed, following exposure to NR e-vapor, *C. albicans* cells exhibited significantly (*p* < 0.01) higher levels of chitin than the control (Figure 3). However, chitin expression was greater in CCS-exposed *C. albicans* than it was in NR and NF e-vapor-exposed cells.

This is the first study to report this modulatory effect of e-cigarette vapor on *C. albicans* chitin content. Similar observations were reported with standard cigarette-exposed *C. albicans*, showing high amounts of chitin in *C. albicans* exposed to cigarette smoke condensate compared to non-exposed *C. albicans* cells [16].

Cell wall proteins, including chitin, are known to be involved in sensing stressful agents, such as changes in carbon source [21]. When exposed to e-vapor, *C. albicans* may consider this contact to be an abnormal situation, thereby promoting chitin production as a protective pathway against the possible deleterious effects of the e-vapor. Indeed, studies showed that *C. albicans* exposed to antifungal molecules increased chitin production to overcome the impact of the drug [16,22]. *C. albicans* may develop a resistance mechanism against CCS and e-cigarette vapor through increased expression of chitin, as is reported in the present study. This may translate to a clinical impact for e-cigarette users.

### 3.3. E-Vapor-Exposed C. albicans Displayed an Increase in Hyphal Length

The ability of *C. albicans* to grow as yeast cells, pseudohyphae, and hyphae is a pivotal aspect of its capacity to move from the commensal to the pathologic phenotype. It has been demonstrated that *C. albicans* virulence can be altered by manipulating the morphology of the yeast [23]. Various forms of *C. albicans* have been found in both infected tissues and biofilms, which suggests a role for each form during infection [24]. As e-cigarettes were capable of promoting *C. albicans* growth, we sought to determine whether e-cigarettes could modulate *C. albicans* morphology.

**Figure 4:**
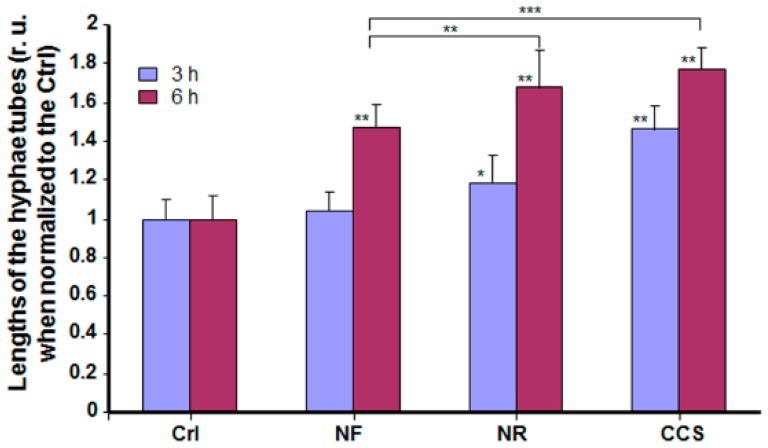
E-cigarette vapor increased the hyphal length of *C. albicans* cultured under cell morphology transition conditions. *C. albicans* cells were exposed or not to CCS, NF e-vapor, or NR e-vapor, then cultured at 37 °C in the presence of 10% fetal calf serum. Hyphal tube length was measured after 3 and 6 h using NIH-ImageJ software (*n* = 5). * *p* < 0.05; ** *p* < 0.01, *** *p* < 0.001 (r. u = relative unit). Free asterisks refer to the statistical difference when comparing exposed (e-vapors/CCS) to non-exposed (Ctrl) *C*. *albicans*. Bars with asterisks show the comparison of NF to NR, and NF to CCS.

Our findings show that hyphal length was significantly higher in e-vapor-exposed cultures compared to the controls. As shown in Figure 4, longer hyphal tubes were observed at 3 and 6 h in the NR e-vapor-exposed and CCS-exposed *C. albicans* cultures. It is important to note that both the NR e-vapor and CCS induced a significantly (*p* < 0.05) longer hyphal size, even at 3 h of incubation, compared to that observed in non-exposed controls. Hyphal tubes were more visible after 6 h of incubation.

Hyphae are considered necessary for *C. albicans* to invade its host [23]. Increased *C. albicans* growth and hyphal length following exposure to e-cigarette vapor may result in oral health issues. Indeed, smokers are reportedly more prone to caries [2], periodontal disease [25], and candidiasis [3]. As e-cigarettes promoted *C. albicans* overgrowth and morphological changes, this may translate to an increased risk of candidiasis and periodontitis in e-cigarette users.

### 3.4. E-Vapor-Exposed C. albicans Expressed High Virulent Gene Levels

**Figure 5:**
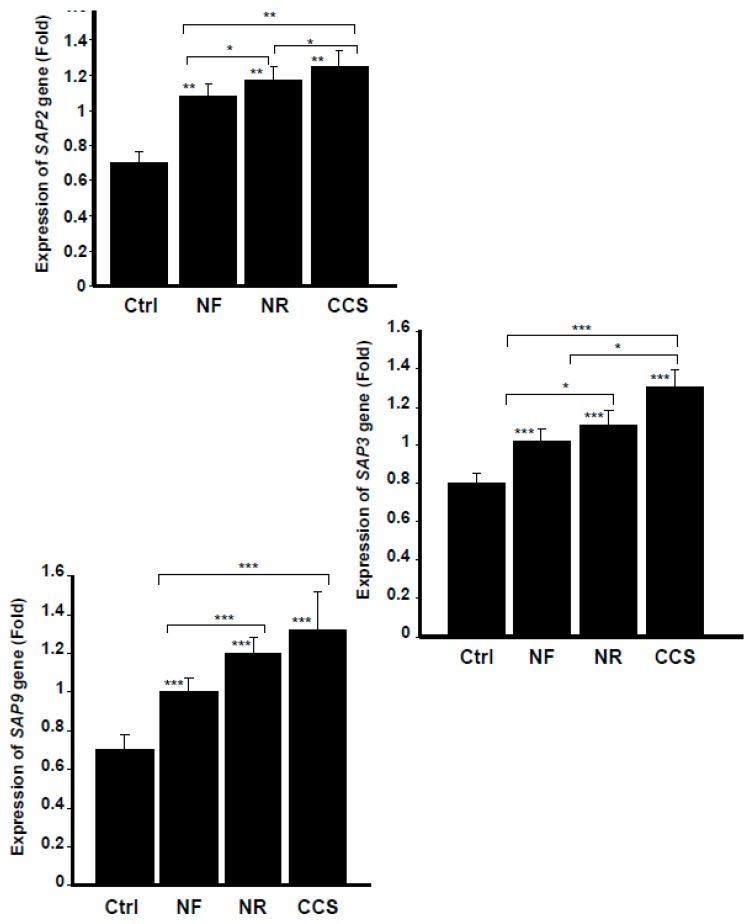
E-cigarette vapor increased the expression of secreted aspartyl proteinases *SAPs 2*, *3*, and *9*. *C. albicans* cells were exposed or not twice a day for 15 min to CCS, NF e-vapor, or NR e-vapor, then incubated for 16 h at 37 °C prior to the extraction of total RNA and analysis by qRT-PCR (*n* = 5). The expression was normalized to the GAPDH (housekeeping gene). Statistical significance was obtained by comparing the cells exposed to CCS or to NR or NF e-vapor and those of the control (non-exposed cells). * *p* < 0.05; ** *p* < 0.01; *** *p* < 0.001. Free asterisks refer to the statistical difference when comparing exposed (e-vapors/CCS) to non-exposed (Ctrl) *C. albicans*. Bars with asterisks show the comparison of NF to NR, NR to CCS, and NF to CCS.

*Candida* growth and morphological change are controlled by various genes [26]. Among these is the *SAP* gene family, known to promote *C. albicans* adhesion, growth, and biofilm formation [27]. Our results show that *C. albicans* exposed to NR e-cigarette vapor expressed a high level of *SAP2*, compared to that observed in the non-exposed culture (Figure 5). *SAP2* mRNA levels were also higher with exposure to NF e-vapor than in the non-exposed cultures (Figure 5). However, the effects of NR and NF e-vapor on *SAP2* gene expression were lower than those recorded for CCS (Figure 5). *SAP2* is associated with *C. albicans* growth and the yeast forms of *C. albicans* [28] and is also essential for mucosal infections [29]. As the e-cigarettes increased *SAP2* expression, this may explain the growth of *C. albicans* observed following its exposure to NR and NF e-vapor and suggests that e-vapor-exposed *C. albicans* could be virulent in smokers.

*SAP3* gene was another aspartyl proteinase modulated through the exposure of *C. albicans* to cigarette smoke and e-vapor. As shown in Figure 5, compared to the non-exposed cultures, NR e-vapor-exposed *C. albicans* expressed a high level of *SAP3* mRNA. It should also be noted that NF e-cigarettes also increased *SAP3* gene expression by *C. albicans*, compared to that observed in the control. However, the effect on *SAP3* expression was greater with the NR e-vapor than with the NF e-vapor, and was greater with CCS than with either e-cigarettes or the control (Figure 5).

As *SAP3* was shown to regulate *C. albicans* growth and phenotypic switching [30], the modulatory effect of e-cigarettes on *SAP3* expression may be a possible mechanism promoting *C. albicans* pathogenesis. The capacity of *C. albicans* to switch reversibly between the white phenotype and the opaque phenotype is required for the yeast to mate. *C. albicans* switching also promotes the yeast’s dissemination, causing systemic candidiasis [31]. We thus suggest that e-cigarettes increase *SAP3* gene expression, which may lead to *C. albicans* switching, and thus to its virulence. Further studies are required to validate this hypothesis.

Our findings also indicate that the e-cigarettes promoted *SAP9* gene expression. Indeed, NR e-cigarette vapor produced a significant (*p* < 0.001) increase of *SAP9* gene expression by *C. albicans* compared to that observed in the non-exposed cultures (Figure 5). NF e-vapor also promoted *SAP9* expression; however, the effect was significantly (*p* < 0.001) greater with NR than with NF e-vapor. It should be noted that when *C. albicans* was exposed to CCS, the expression of *SAP9* was significantly (*p* < 0.001) higher than that observed in the control or with the NR or NF e-vapor (Figure 5). Similar to other secreted aspartic proteases, *SAP9* is associated with the fungal cell wall [27,32] and is reported to upregulate under biofilm-forming conditions [33]. *SAP9* thus contributes to the process of *C. albicans* [34] and the decrease of host innate immunity [35], which may favor the onset of *C. albicans* infection.

### 3.5. E-Vapor-Exposed C. albicans Adhered Better to Gingival Epithelial Cells

**Figure 6:**
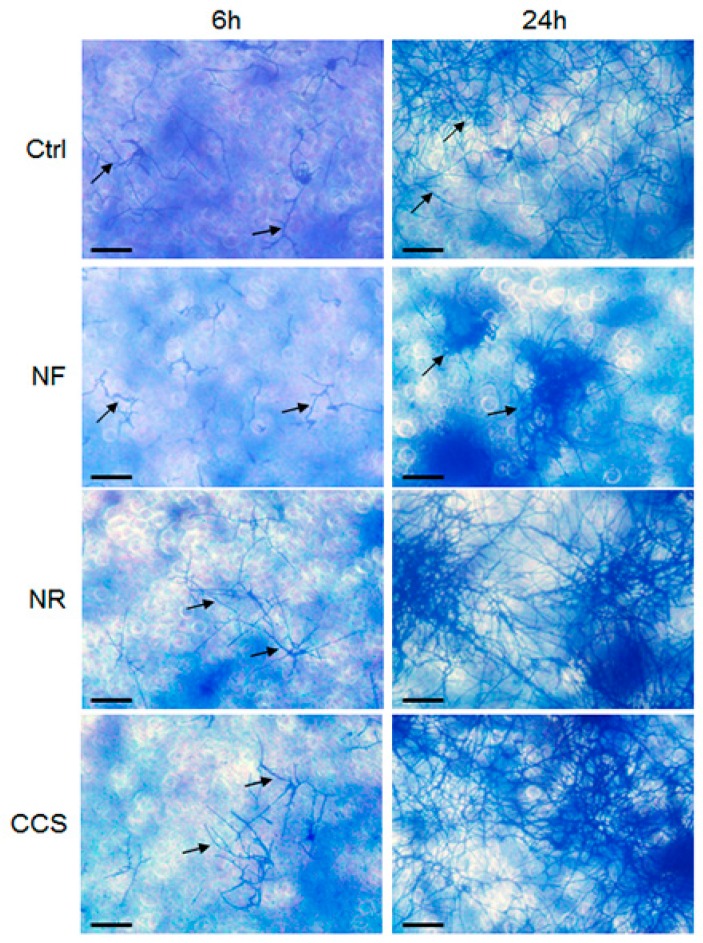
*C. albicans* pre-exposed to e-cigarette vapor adhered better to gingival epithelial cells cultures. Gingival epithelial cells were seeded in 6-well plates and cultured for 24 h. The cell monolayers were then co-cultured with e-vapor pre-exposed *C. albicans*. Adhesion of *C. albicans* to the gingival cells was assessed after 6 and 24 h using the crystal violet staining assay. Representative images are from four independent experiments, with each experiment performed in duplicate. Scale bars = 50 μm.

The effect of e-cigarettes on *C. albicans* growth and *SAP* gene expression raised the following question: How do e-vapor-exposed *C. albicans* cells interact with gingival epithelial cells? Figure 5 shows that *C. albicans* adhesion to the epithelial cell monolayer culture was greater following exposure to NR e-vapor than it was in the control. On the other hand, the adhesion observed of NR e-vapor-exposed *C. albicans* to epithelial cells was lower than that observed in CCS-exposed cells. It should be noted that at 24 h, *C. albicans* adopted the hyphal form, covering a larger area of the epithelial monolayer culture (Figure 6). The cell density of the hyphae in the NR e-vapor-exposed *C. albicans* adhering to the epithelial cell culture was greater than that observed with the non-exposed *C. albicans*. This is the first study demonstrating the effect of e-cigarettes in modulating *C. albicans* adhesion to epithelial cells. This may be explained by an increased contact of *C. albicans* with epithelial cells through cell wall proteins, as chitin content increased in the e-vapor-exposed *C. albicans* (Figure 3). As e-cigarettes were shown to promote *C. albicans* growth, an increase in chitin content and *SAP* gene expression, we performed an indirect interaction study between e-vapor pre-exposed *C. albicans* and gingival epithelial cells using a trans-well culture system.

### 3.6. Crosstalk Interactions between e-Vapor-Exposed C. albicans and Epithelial Cells Promoted the Yeast’s Growth and Morphological Changes

**Figure 7:**
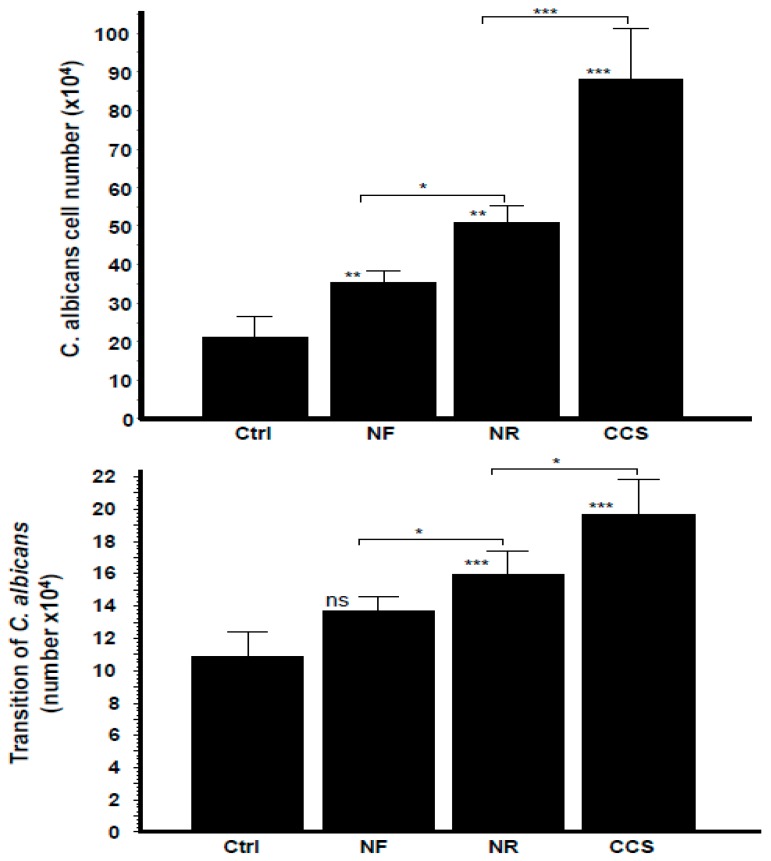
Growth and transition of *C. albicans* pre-exposed to e-cigarette vapor then co-cultured with gingival epithelial cells. *C. albicans* cells were exposed twice a day for 15 min to CCS, NR e-vapor, or NF e-vapor, followed by co-culture with gingival epithelial cells in a trans-well culture system. After 24 h, the *C. albicans* cells in the upper chamber were collected and used to determine their growth (upper figure) and transition (lower figure) by optical microscope analysis (*n* = 4). * *p* < 0.05; ** *p* < 0.01; *** *p* < 0.001. Free asterisks refer to the statistical difference when comparing exposed (e-vapors/CCS) to the non-exposed (Ctrl) *C. albicans*. Bars with asterisks showed the comparison of NF to NR, and NR to CCS.

As shown in Figure 7, *C. albicans* growth and hyphal morphological changes were significantly increased in both the e-vapor and CCS pre-exposed *C. albicans* co-cultured with gingival epithelial cells, with observed growth approximately two-folds greater with NR e-vapor, compared to the control (Figure 7). NR e-vapor-exposed *C. albicans* co-cultured with epithelial cells also showed a significant (*p* < 0.01) growth increase compared to the control. However, the greatest growth increase was obtained following *C. albicans* exposure to CCS and subsequent co-culture with epithelial cells for 24 h. *C. albicans* morphological change from blastospore to hyphal form was also modulated by exposure to e-vapor or to CCS and co-culture with gingival epithelial cells. As shown in Figure 7, both the NR and NF e-vapor-exposed *C. albicans* co-cultured with epithelial cells recorded a significant high transition against the control. Furthermore, the effect of NR e-vapor was significantly greater than that of NF e-vapor, while CCS represented the agent producing the greatest transition of *C. albicans* following co-culture with gingival epithelial cells (Figure 7). This is in agreement with previous studies showing increased bacterial adhesion to and colonization on epithelial cells in the presence of cotinine or nicotine [36]. The effect of e-cigarette vapor and CCS on *C. albicans* growth and form changing when in contact with epithelial cells may be due to the elevated expression of chitin, as this cell wall protein increased after exposure to e-cigarette vapor (Figure 3). The high level of chitin could thus play a role in promoting the interaction of *C. albicans* with the host cells, as previously reported [16,37]. As *C. albicans* exposed to e-vapor/CCS and co-cultured with gingival epithelial cells showed increased growth and morphological change, we suggest that this may affect epithelial cell behaviors.

### 3.7. E-Vapor-Exposed C. albicans Promoted Morphological Changes in Epithelial Cells and Reduced Their Growth

**Figure 8:**
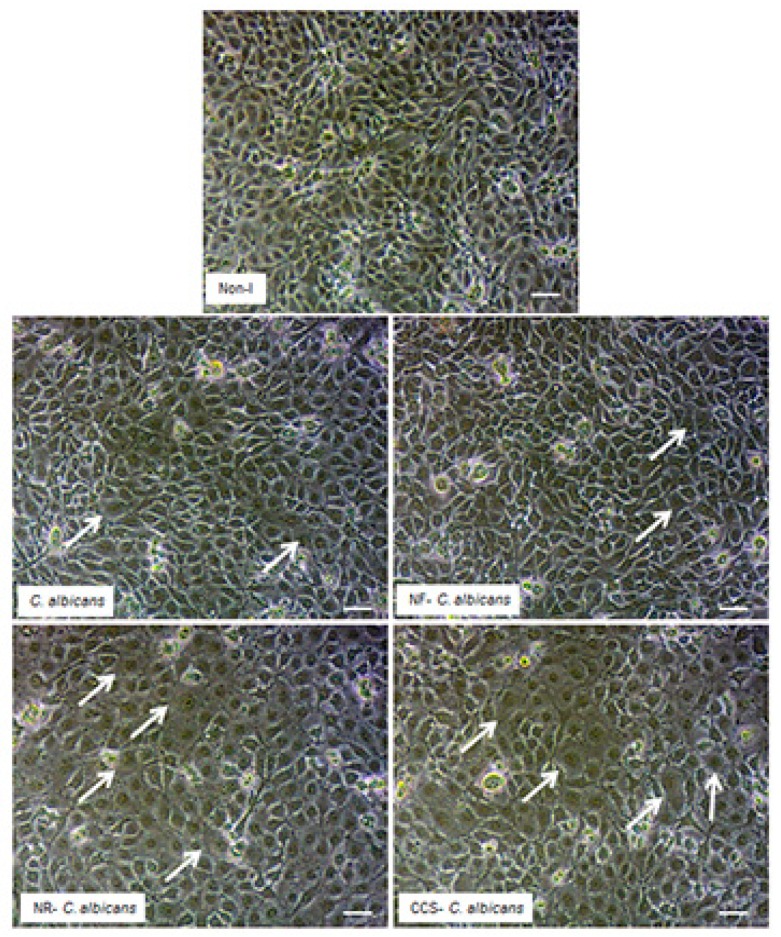
*C. albicans* pre-exposed to e-cigarette vapor promoted gingival epithelial cell differentiation. Gingival epithelial cell monolayers were co-cultured with *C. albicans* pre-exposed to e-vapor twice a day for 15 min. Following co-culture for 24 h in a trans-well culture system, the epithelial cell monolayers in the lower culture chambers were observed under an inverted optical microscope and photographed. Representative images are from four independent experiments, with each experiment performed in duplicate. Arrows indicate the differentiated cells. Scale bars = 50 μm.

**Figure 9:**
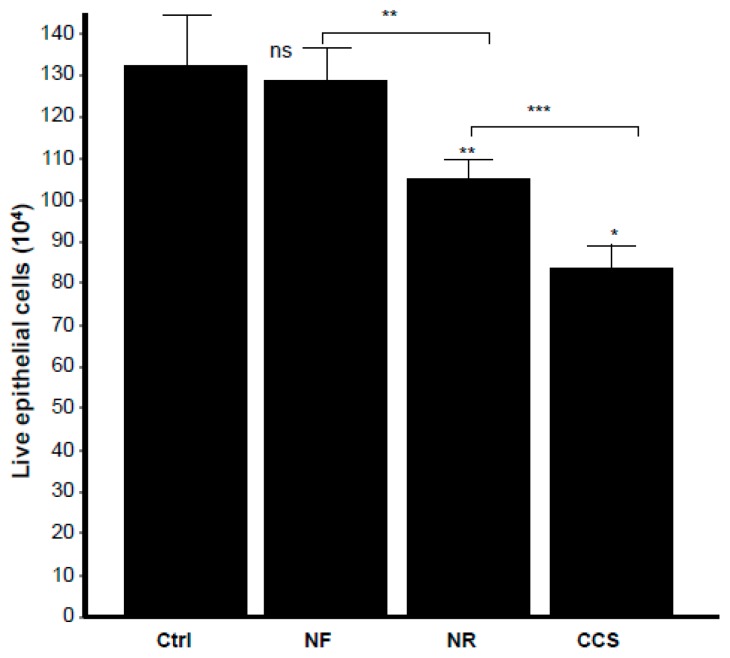
E-vapor pre-exposed *C. albicans* decreased gingival epithelial cell viability. Gingival epithelial cells were co-cultured for 24 h in the presence of e-vapor pre-exposed *C. albicans*. Epithelial cells were then detached, and their viability was determined by trypan blue exclusion assay (*n* = 4). Statistical significance was obtained by comparing the cells exposed to CCS, NR e-vapor, or NF e-vapor with those of the control (non-exposed cells). * *p* < 0.05; ** p < 0.01; *** *p* < 0.001; ns = non-significant as compared to the control. Free asterisks refer to the statistical difference when comparing exposed (e-vapors/CCS) to the non-exposed (Ctrl) *C. albicans*. Bars with asterisks show the comparison of NF to NR, NR to CCS.

**Figure 10:**
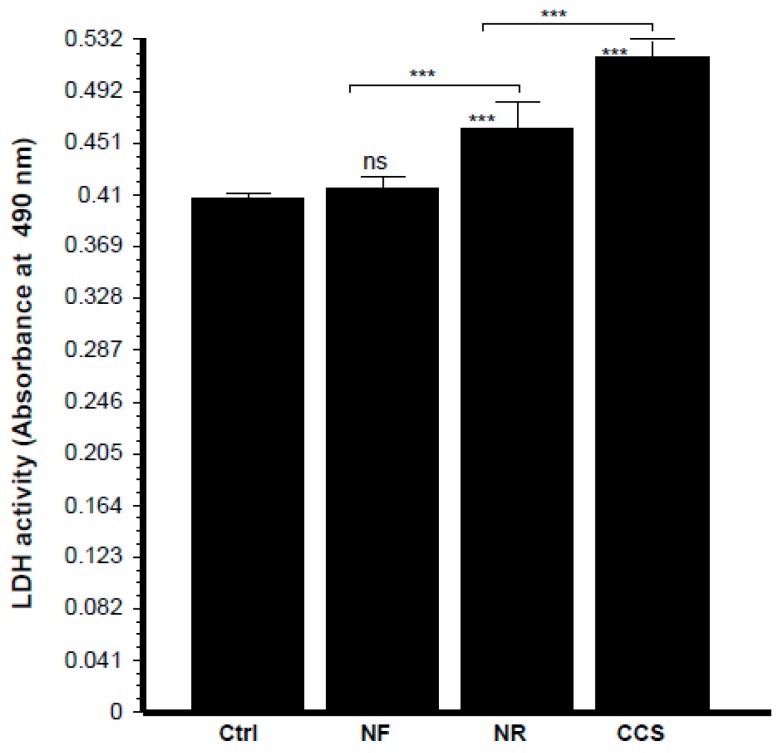
Epithelial cells co-cultured with e-vapor pre-exposed *C. albicans* displayed high levels of lactate dehydrogenase (LDH) activity. Gingival epithelial cells were co-cultured for 24 h in the presence of e-vapor pre-exposed *C. albicans*. Culture supernatants were collected and used to measure LDH activity, as described in the Materials and Methods section (*n* = 4). Statistical significance was obtained by comparing the cells exposed to CCS, NR e-vapor, or NF e-vapor with those of the control (non-exposed cells). *** *p* < 0.001; ns = non-significant. Free asterisks refer to the statistical difference when comparing exposed (e-vapors/CCS) to non-exposed (Ctrl) *C. albicans*. Bars with asterisks show the comparison of NF to NR, NR to CCS.

Microscopic observations of the epithelial cell monolayer following culture in the presence of either e-vapor-exposed or CCS-exposed *C. albicans* revealed the presence of differentiated epithelial cells (Figure 8, arrows). These large-sized cells were characterized by a wide and faint nucleus, large cytoplasm, and the presence of vacuoles in the culture being pulsed with NR e-vapor-exposed *C. albicans*. Fewer differentiated cells were observed with the NF e-vapor and in the control compared to the NR e-vapor and CCS conditions. The greatest number of differentiated cells was observed with exposure to CCS (Figure 8). Therefore, even with indirect contact, e-vapor-exposed *C. albicans* exerted some adverse effects on the gingival epithelial cells by modulating their cell shape. Cell sensitivity to stress leading to morphological changes was also reported with primary human gingival epithelial cells exposed to e-cigarettes [20], and skin keratinocytes exposed to ultraviolet radiation [38]. The morphological changes observed following epithelial culture in the presence of e-vapor- or CCS-exposed *C. albicans* are supported by the reduction in epithelial cell growth. As shown in Figure 9, the viable epithelial cell number decreased significantly (*p* < 0.05) in the cultures pulsed with e-vapor- or CCS-exposed *C. albicans*. Indeed, the number of viable cells dropped from 13 × 10^5^ cells in the control to 10 × 10^5^ with the NR e-vapor and 8 × 10^5^ with CCS. Furthermore, the decrease in epithelial cell viability was accompanied by an increase in LDH activity. As shown in Figure 10, high levels of LDH activity were recorded by epithelial cells pulsed with NR e-vapor-exposed *C. albicans*.

This study is the first to demonstrate the possible adverse effects of e-cigarette-exposed *C. albicans* on gingival epithelial cells. It suggests that e-cigarette vapor may enhance the capacity of *C. albicans* to evade epithelial cell defenses by promoting overgrowth and transition (Figure 7). E-cigarettes have already been shown to produce negative effects on different cell types, including gingival fibroblasts [39], epithelial cells [20], endothelial cells [40], and osteoblasts [41]. The present study supports existing data by showing that e-cigarettes affect oral microbial behaviors by stimulating their pathogenesis through overgrowth, transition, and the expression of virulent genes, such as *SAPs*.

## 4. Conclusions

This study demonstrates that e-cigarettes with or without nicotine promoted the growth and hyphal length of *C. albicans*, and that both nicotine-free and nicotine-rich e-cigarettes increased the expression of different *SAP* genes, such as *SAP2*, *SAP3*, and *SAP9*, which are known to contribute to *C. albicans* growth and virulence. Our findings also confirm that co-culture with e-vapor-exposed *C. albicans* increased gingival epithelial cell differentiation and reduced their growth. The co-culture showed even higher growth and morphological change of e-vapor-exposed *C. albicans* than when placed in indirect contact with epithelial cells, compared to that observed with non-exposed *C. albicans*. Overall, results show the contribution of e-cigarette exposure to *C. albicans* overgrowth, and virulent genes expression, which could cause oral candidiasis in individuals carrying *C. albicans* and using e-cigarettes.

## Figures and Tables

**Table 1:** Primer sequences used for the qRT-PCR. Primers were optimized previously [10,15].

Gene	Primer Sequence (5′ à 3′)	Tm-m	Tm-Exp	Amp Size (Bp)
*ACT1*	Forward: GACAATTTCTCTTTCAGCACTAGTAGTGA	57 °C	60 °C	87
	Reverse: GCTGGTAGAGACTTGACCAACCA	59 °C	60 °C	
*SAP2*	Forward: TCCTGATGTTAATGTTGATTGTCAAG	54 °C	60 °C	82
	Reverse: TGGATCATATGTCCCCTTTTGTT	54 °C	60 °C	
*SAP3*	Forward: GGACCAGTAACATTTTTATGAGTTTTGAT	55 °C	60 °C	87
	Reverse: TGCTACTCCAACAACTTTCAACAAT	56 °C	60 °C	
*SAP9*	Forward: ATTTACTCCACAGTTTATCACTGAAGGT	57 °C	60 °C	86
	Reverse: CCACAAGAACCACCCTCAGTT	57 °C	60 °C	

Tm-m = melting temperature from the manufacturer; Tm-Exp = experimental melting temperature optimized with our primers.

## References

[1] Feldman C., Anderson R. (2013). Cigarette smoking and mechanisms of susceptibility to infections of the respiratory tract and other organ systems. J. Infect..

[2] Vellappally S., Fiala Z., Smejkalová J., Jacob V., Shriharsha P. (2007). Influence of tobacco use in dental caries development. Cent. Eur. J. Public Health.

[3] Baboni F.B., Barp D., Izidoro A.C., Samaranayake L.P., Rosa E.A. (2009). Enhancement of *Candida albicans* virulence after exposition to cigarette mainstream smoke. Mycopathologia.

[4] Bagaitkar J., Williams L.R., Renaud D.E., Bemakanakere M.R., Martin M., Scott D.A., Demuth D.R. (2009). Tobacco-induced alterations to *Porphyromonas gingivalis*-host interactions. Environ. Microbiol..

[5] Canabarro A., Valle C., Farias M.R., Santos F.B., Lazera M., Wanke B. (2013). Association of subgingival colonization of *Candida albicans* and other yeasts with severity of chronic periodontitis. J. Periodontal Res..

[6] Dahlen G. (1993). Role of suspected periodontopathogens in microbiological monitoring of periodontitis. Adv. Dent. Res..

[7] Karkowska-Kuleta J., Bartnicka D., Zawrotniak M., Zielinska G., Kieronska A., Bochenska O., Ciaston I., Koziel J., Potempa J., Baster Z. (2018). The activity of bacterial peptidylarginine deiminase is important during formation of dual-species biofilm by periodontal pathogen *Porphyromonas gingivalis* and opportunistic fungus *Candida albicans*. Pathog. Dis..

[8] Al Mubarak S., Robert A.A., Baskaradoss J.K., Al-Zoman K., Al Sohail A., Alsuwyed A., Ciancio S. (2013). The prevalence of oral Candida infections in periodontitis patients with type 2 diabetes mellitus. J. Infect. Public Health.

[9] Rubio N.A., Puia S., Toranzo S., Brusca M.I. (2015). Fungal invasion of connective tissue in patients with gingival-periodontal disease. Rev. Iberoam. Micol..

[10] Semlali A., Killer K., Alanazi H., Chmielewski W., Rouabhia M. (2014). Cigarette smoke condensate increases *C. albicans* adhesion, growth, biofilm formation, and EAP1, HWP1 and SAP2 gene expression. BMC Microbiol..

[11] Wigginton B., Gartner C., Rowlands I.J. (2017). Is It Safe to Vape? Analyzing online forums discussing e-cigarette use during pregnancy. Womens Health Issues.

[12] Margham J., McAdam K., Forster M., Liu C., Wright C., Mariner D., Proctor C. (2016). Chemical Composition of Aerosol from an E-Cigarette: A Quantitative Comparison with Cigarette Smoke. Chem. Res. Toxicol..

[13] Lerner C.A., Sundar I.K., Watson R.M., Elder A., Jones R., Done D., Kurtzman R., Ossip D.J., Robinson R., McIntosh S. (2015). Environmental health hazards of e-cigarettes and their components: Oxidants and copper in e-cigarette aerosols. Environ. Pollut..

[14] Lerner C.A., Rutagarama P., Ahmad T., Sundar I.K., Elder A., Rahman I. (2016). Electronic cigarette aerosols and copper nanoparticles induce mitochondrial stress and promote DNA fragmentation in lung fibroblasts. Biochem. Biophys. Res. Commun..

[15] Belmadani A., Semlali A., Rouabhia M. (2018). Dermaseptin-S1 decreases *Candida albicans* growth, biofilm formation and the expression of hyphal wall protein 1 and aspartic protease genes. J. Appl. Microbiol..

[16] Alanazi H., Semlali A., Perraud L., Chmielewski W., Zakrzewski A., Rouabhia M. (2014). Cigarette smoke-exposed *Candida albicans* increased chitin production and modulated human fibroblast cell responses. Biomed. Res. Int..

[17] Livak K.J., Schmittgen T.D. (2001). Analysis of relative gene expression data using real-time quantitative PCR and the 2(-Delta Delta C(T)) method. Methods.

[18] Imamura K., Kokubu E., Kita D., Ota K., Yoshikawa K., Ishihara K., Saito A. (2016). Role of mitogen-activated protein kinase pathways in migration of gingival epithelial cells in response to stimulation by cigarette smoke condensate and infection by *Porphyromonas gingivalis*. J. Periodontal Res..

[19] Camenga D.R., Tindle H.A. (2018). Weighing the risks and benefits of electronic cigarette use in high-risk Populations. Med. Clin. N. Am..

[20] Rouabhia M., Park H.J., Semlali A., Zakrzewski A., Chmielewski W., Chakir J. (2017). E-Cigarette vapor induces an apoptotic response in human gingival epithelial cells through the caspase-3 pathway. J. Cell. Physiol..

[21] Brown A.J.P., Budge S., Kaloriti D., Tillmann A., Jacobsen M.D., Yin Z., Ene I.V., Bohovych I., Sandai D., Kastora S. (2014). Stress adaptation in a pathogenic fungus. J. Exp. Biol..

[22] Walker L.A., Munro C.A., De Bruijn I., Lenardon M.D., McKinnon A., Gow N.A.R. (2008). Stimulation of chitin synthesis rescues Candida albicans from echinocandins. PLoS Pathog..

[23] Saville S.P., Lazzell A.L., Monteagudo C., Lopez-Ribot J.L. (2003). Engineered control of cell morphology in vivo reveals distinct roles for yeast and filamentous forms of *Candida albicans* during infection. Eukaryot. Cell.

[24] Hirakawa M.P., Martinez D.A., Sakthikumar S., Anderson M.Z., Berlin A., Gujja S., Zeng Q., Zisson E., Wang J.M., Greenberg J.M. (2015). Genetic and phenotypic intra-species variation in *Candida albicans*. Genome Res..

[25] Vogtmann E., Graubard B., Loftfield E., Chaturvedi A., Dye B.A., Abnet C.C., Freedman N.D. (2017). Contemporary impact of tobacco use on periodontal disease in the USA. Tob. Control.

[26] Mathe L., Van Dijck P. (2013). Recent insights into *Candida albicans* biofilm resistance. Curr. Genet..

[27] Monod M., Hube B., Hess D., Sanglard D. (1998). Differential regulation of SAP8 and SAP9, which encode two new members of the secreted aspartic proteinase family in *Candida albicans*. Microbiology.

[28] Cavalcanti Y.W., Wilson M., Lewis M., Del-Bel-Cury A.A., da Silva W.J., Williams D.W. (2016). Modulation of *Candida albicans* virulence by bacterial biofilms on titanium surfaces. Biofouling.

[29] Naglik J.R., Moyes D., Makwana J., Kanzaria P., Tsichlaki E., Weindl G., Tappuni A.R., Rodgers C.A., Woodman A.J., Challacombe S.J. (2008). Quantitative expression of the *Candida albicans* secreted aspartyl proteinase gene family in human oral and vaginal candidiasis. Microbiology.

[30] Morrow B., Srikantha T., Anderson J., Soll D.R. (1993). Coordinate regulation of two opaque-phase-specific genes during white-opaque switching in *Candida albicans*. Infect. Immun..

[31] Solis N.V., Park Y.N., Swidergall M., Daniels K.J., Filler S.G., Soll D.R. (2018). *Candida albicans* white-opaque switching influences virulence but not mating during oropharyngeal candidiasis. Infect. Immun..

[32] Schild L., Heyken A., de Groot P.W., Hiller E., Mock M., de Koster C., Horn U., Rupp S., Hube B. (2011). Proteolytic cleavage of covalently linked cell wall proteins by *Candida albicans* SAP9 and SAP10. Eukaryot. Cell.

[33] Joo M.Y., Shin J.H., Jang H.C., Song E.S., Kee S.J., Shin M.G., Suh S.P., Ryang D.W. (2013). Expression of *SAP5* and *SAP9* in *Candida albicans* biofilms: Comparison of bloodstream isolates with isolates from other sources. Med. Mycol..

[34] Albrecht A., Felk A., Pichova I., Naglik J.R., Schaller M., de Groot P., Maccallum D., Odds F.C., Schäfer W., Klis F. (2006). Glycosylphosphatidyinositol-anchored proteases of *Candida albicans* target proteins necessary for both cellular processes and host-pathogen interactions. J. Biol. Chem..

[35] Rapala-Kozik M., Bochenska O., Zawrotniak M., Wolak N., Trebacz G., Gogol M., Ostrowska D., Aoki W., Ueda M., Kozik A. (2015). Inactivation of the antifungal and immunomodulatory properties of human cathelicidin LL-37 by aspartic proteases produced by the pathogenic yeast *Candida albicans*. Infect. Immun..

[36] Teughels W., Van Eldere J., van Steenberghe D., Cassiman J.J., Fives-Taylor P., Quirynen M. (2005). Influence of nicotine and cotinine on epithelial colonization by periodontopathogens. J. Periodontol..

[37] Plaine A., Walker L., Da Costa G., Mora-Montes H.M., McKinnon A., Gow N.A., Gaillardin C., Munro C.A., Richard M.L. (2008). Functional analysis of *Candida albicans* GPI-anchored proteins: Roles in cell wall integrity and caspofungin sensitivity. Fungal Genet. Biol..

[38] Pâquet I., Chouinard N., Rouabhia M. (1996). Cutaneous cell and extracellular matrix responses to ultraviolet-B irradiation. J. Cell. Physiol..

[39] Alanazi H., Park H.J., Chakir J., Semlali A., Rouabhia M. (2018). Comparative study of the effects of cigarette smoke and electronic cigarettes on human gingival fibroblast proliferation, migration and apoptosis. Food Chem. Toxicol..

[40] Anderson C., Majeste A., Hanus J., Wang S. (2016). E-Cigarette aerosol exposure induces reactive oxygen species, DNA damage, and cell death in vascular endothelial cells. Toxicol. Sci..

[41] Rouabhia M., Alanazi H., Park H.J., Gonçalves R.B. (2018). Cigarette smoke and E-cigarette vapor dysregulate osteoblast interaction with titanium dental implant surface. J. Oral Implantol..

